# Long non-coding RNA MIAT promotes gastric cancer growth and metastasis through regulation of miR-141/DDX5 pathway

**DOI:** 10.1186/s13046-018-0725-3

**Published:** 2018-03-14

**Authors:** Min Sha, Mei Lin, Jia Wang, Jun Ye, Jie Xu, Ning Xu, Junxing Huang

**Affiliations:** 1 Institute of Clinical medicine, Taizhou people’s Hospital affiliated of Nantong University of medicine, Taizhou, China; 2 Department of Reproductive Medicine, Taizhou people’s Hospital affiliated of Nantong University of medicine, Taizhou, China; 3 Department of Gastroenterology, Taizhou people’s Hospital affiliated of Nantong University of medicine, Taizhou, China; 4 Institute of Oncology, Taizhou people’s Hospital affiliated of Nantong University of medicine, 210 Yingchun, Taizhou, Jiangsu Province 225300 China

**Keywords:** Gastric cancer, MIAT, miR-141, DDX5

## Abstract

**Background:**

The objective of this study was to investigate the role and mechanism of long non-coding RNA MIAT in gastric cancer (GC).

**Methods:**

Real-time PCR was used to determine MIAT level in 120 GC tissues, and in two gastric cancer cell lines. The clinicopathological characteristics of MIAT in GC patients were analyzed. Small interfering RNA specific for MIAT (si-MIAT) and lentivector for si-MIAT was performed to down-regulate MIAT expression in GC cells and in animal tumor model, respectively. The interaction of MIAT and miR-141 was measured by RNA pull-down assay and RNA immunoprecipitation. The biological function of si-MIAT on GC cell growth and metastasis were explored through flow cytometry assay, invasion and migration assay in vitro.

**Results:**

MIAT was highly expressed in GC tissues and cell lines and correlated with differentiation degree, TNM stage, distant metastasis, and lymph node metastasis. MIAT knockdown inhibited GC growth and metastasis both in vitro and in vivo. Furthermore, MIAT acted as miR-141 sponge and regulated its target gene DDX5 expression. In BGC-823 and MGC-803 cells with si-MIAT, DDX5 overexpression resulted in an increase of cell proliferation, migration and invasion.

**Conclusions:**

Our data indicated that MIAT played an oncogenic role in GC growth and metastasis, and could serve as a novel molecular target for treating GC.

**Electronic supplementary material:**

The online version of this article (10.1186/s13046-018-0725-3) contains supplementary material, which is available to authorized users.

## Background

Gastric cancer (GC), one of the most common cancers, is the major cause of global cancer mortality, especially in Eastern Asia [1]. Although several improvements have been made in diagnosis and therapy of patients with early stages of GC, it is extremely poor to treat GC at an advanced stage. Proliferation and metastasis are two important factors which are closely linked with poor prognosis and death in GC [2, 3]. In recent years, molecules closely related to cell proliferation and metastasis have been studied as targeted agents for GC therapy [4]. However, the molecular mechanism of GC cell growth and metastasis remains unclear. Therefore, it is urgent to make clear the key molecule involved in growth, invasion and migration of GC cells.

The DEAD-box RNA helicase 5 (DDX5), an ATP-dependent DEAD-box RNA helicase, acts as a transcriptional co-activator of several cancer-associated transcription factors and plays an important role in transcription initiation [5]. As an oncogene, DDX5 is overexpressed in a variety of tumors and contributes to promoting cancer cell proliferation and metastasis [6, 7]. In GC, high DDX5 expression was associated with advanced clinical stage [8]. Furthermore, overexpression of DDX5 promoted GC cell growth in vitro and in vivo, while down-regulation of DDX5 expression could inhibit GC cell growth [8].

Long non-coding RNA MIAT (Myocardial infarction associated transcript) is mainly expressed in the nucleus and highly conserved among mammalian species [9]. In recent years, MIAT has been confirmed to be involved in development and progression of several cancers. MIAT had high expression in malignant mature B cells, which contributed the progression of chronic lymphocytic leukemias [10]. Besides, MIAT was associated with the development of lung adenocarcinoma [11]. Furthermore, MIAT exerted oncogenic effect on neuroendocrine prostate cancer [12]. However, the exact role and mechanism of MIAT on GC cell proliferation and metastasis remains unclear.

MicroRNA-141 (miR-141) has been shown to be down-regulated in GC tissues and its low level was obviously associated with poor differentiation, metastasis, and advanced TNM stage of patients with GC [13]. Further study demonstrated that over-expression of miR-141 could inhibit GC proliferation and metastasis, while knockdown of miR-141 could promote GC growth in vitro and in vivo [14]. Therefore, miR-141 might serve as a prognostic factor and therapeutic candidate in GC patients.

In the present study, we found that MIAT expression was significantly increased in GC tissues and cells. Knockdown of MIAT led to impaired proliferation and metastasis of GC cells. We also observed that MIAT could interact with miR-141. The down-regulation of MIAT resulted in the increase of miR-141 expression and the decrease of DDX5 expression. Taken together, our data revealed an oncogenic role of the long noncoding RNA MIAT in GC and its role in regulating DDX5 expression.

## Methods

### Patients and tissue samples

Paired gastric cancer and normal gastric tissue were obtained between 2014 and 2016 from 120 patients (age: 28-85 years, median 60.4 years) who underwent primary surgical resection of gastric cancer in Department of Gastroenterology of Taizhou people’s Hospital affiliated of Nantong University of medicine (Taizhou, China). Follow-up information was obtained by reviewing patients’ medical records. None of the patients received radiotherapy or chemotherapy before surgical resection. All these tissue samples were immediately frozen in liquid nitrogen and stored at − 80 °C until total RNA was extracted.

### Cell culture

Human gastric adenocarcinoma cell line BGC-823, gastric mucinous adenocarcinoma cell line MGC-803 were purchased from the Shanghai Institute of Cytobiology in Chinese Academy of Sciences. Human normal gastric epithelial cell line GES-1 was obtained from the Institute of Oncology in Beijing University. Cells were cultured in Dulbecco’s modified Eagle’s medium (DMEM; Gibco-BRL; Rockville, MD, USA) with 10% Fetal bovine serum (FBS; PAA Laboratories; GmbH, Linz, Austria) at 37 °C in a humidified incubator containing 5% CO_2_.

### Real-time PCR assay

Total RNA of frozen tissues and cells were extracted using TRIzol reagent (Invitrogen, Grand Island, NY, USA) according to the manufacturer’s protocol. MIAT expression and mRNA level of DDX5 and PLK1 were quantified by real-time PCR using a LightCycler480 II Sequence Detection System (Roche, Basel, Switzerland). Sequences of the primers were available in Additional file 1: Table S1. GAPDH and U6 were used as internal standard using 2^-∆∆Ct^ method.

### Western blotting

Total protein from tissue and cells were extracted in lysis buffer (Vazyme Biotech, Nanjing, China). Protein concentrations were determined using the Bio-Rad Protein Assay. Western blot analysis was performed as described [15]. Individual immunoblots were probed with primary antibodies, anti-DDX5 antibody (Cell Signaling Technology; Beverly, MA, USA; diluted 1:1000) and anti-β-Actin antibody (Santa Cruz Biotechnologies; Santa Cruz, CA, USA; diluted 1:3000).

### Small interfering RNA

Small interfering RNA specific for MIAT (si-MIAT) and control siRNA (si-control) was synthesized (Ribobio, Guangzhou, China) and transfected using Lipofectamine 2000 in gastric cells. The sequences of siRNA were on Additional file 1: Table S1.

### Plasmid

The plasmid expressing DDX5 (pcDNA-DDX5) was constructed as previous description [16]. BGC-823 cells were transfected with si-MIAT-1 and pcDNA-DDX5 for 48 h using Lipofectamine 2000 following the manufacturer’s protocol.

### CCK-8 assay

Cell viability was measured by CCK-8 assay. Briefly, cells were plated in 96-well plates at a density of 3000 cells / well. Then, 10 μl of CCK-8 solution (Sigma, MO, USA) was added to each well and incubated at 37 °C for 2 h, and then the absorbance was measured at 450 nm. The experiment was performed independently in triplicate.

### Flow cytometry analysis

Cell cycle was analyzed by flow cytometry analysis. Before transfection, serum was deprived for 24 h to synchronize cell cycle. Then, serum was added back, and si-MIAT or si-control transfected gastric cells were cultured in six-well plates. After transfection for 24 h, the cells were fixed with 80% cooled ethanol, and incubated with 0.5% Triton X-100 solution containing 1 mg / mL RNase A at 37 °C for 30 min. Next, PI (Sigma, MO, USA) was added into the wells at a final concentration of 50 μg / mL. Cellular DNA content was analyzed by a FACS (Becton Dickin-son). Data were processed using Cell-Quest software (Becton Dickinson).

Apoptosis was also analyzed by flow cytometry analysis. The si-MIAT or si-control transfected gastric cells were cultured in six-well plates for 48 h. The cells were harvested by trypsinization. Following double staining with FITC-annexin V and propidium iodide (PI), the cells were analyzed using flow cytometry (FACScan; BD Biosciences, San Jose, CA).

### Lentivirus infection

BGC-823 cells were stably transduced with MIAT-set small interfering RNA (si-MIAT) Lentivector as well as control vector (GENECHEM, Shanghai, China). Transfection of BGC-823 cells was done at a 10-fold MOI (multiplicity of infection) virus particle concentration. MIAT expression was determined by Real-time PCR assay.

### Invasion and migration assay

For the migration assays, 1 × 10^5^ gastric cancer cells in serum-free media were placed into the upper chamber of a Transwell insert. For the invasion assays, gastric cancer cells in serum-free medium were placed into the upper chamber of an insert coated with Matrigel. Medium containing 10% FBS was added to the lower chamber. After incubation for 16 h, the cells remaining on the upper membrane were removed with cotton wool. Gastric cancer cells that had migrated or invaded through the membrane were fixed in methanol, stained with crystal violet (0.04% in water; 100 μl), counted using an inverted microscope and photographed.

### Pull-down assay with biotinylated lncRNA-MIAT DNA probe

MIAT was transcribed from vector pSPT19-MIAT and biotin-labeled with the Biotin RNA Labeling Mix (Roche Diagnostics, Indianapolis, IN) and SP6 RNA polymerase (Roche Applied Science, Basel, Switzerland), and purified with an RNeasy Mini Kit (Qiagen, Valencia, CA). The biotinylated MIAT DNA probe was dissolved in binding and washing buffer, and incubated with Dynabeads M-280 Streptavidin (Invitrogen, CA, USA) at room temperature for 10 min to generate probe-coated beads according to the manufacturer’s protocol. Then, BGC-823 cell lysates were incubated with the probe-coated beads, and the RNA complexes bound to these beads were eluted and extracted for Real-time PCR analysis.

### Pull-down assay with biotinylated miR-141

BGC-823 cells were transiently transfected with biotinylated miR-141, miR-141-Mut (mutation) and negative control (Ribobio, Guangzhou, China), harvested and lysed 48 h after transfection. 50 μL of the samples were aliquoted for input. The remaining lysates were incubated with Dynabeads M-280 Streptavidin (Invitrogen, CA, USA) according to the manufacturer’s protocol. In brief, the washed beads were treated in RNase-free solutions and incubated with equal volume of biotinylated miR-141 for 10 min at room temperature in binding and washing buffer on a rotator. Then, the beads with the immobilized miR-141 fragment were incubated with 10 mM EDTA (pH = 8.2) with 95% formamide at 65 °C for 5 min. The bound RNAs were purified using Trizol for the Real-time PCR analysis.

### RNA immunoprecipitation (RIP)

RIP experiments were performed using the Magna RIP™ RNA-binding protein immunoprecipitation kit (Millipore, Bedford, MA, USA) according to the manufacturer’s instructions. The antibody for RIP assays of AGO2 (Cell Signaling Technology; Beverly, MA, USA) was diluted 1:50. Co-precipitated RNAs were detected by quantitative RT-PCR.

### Luciferase reporter assays

The 3’-UTR of human DDX5 or lncRNA-MIAT was amplified from human genomic DNA and individually inserted into the pmiR-RB-REPORT™ (Ribobio, Guangzhou, China) using the XhoI and NotI sites. Similarly, the fragment of DDX5 or lncRNA-MIAT 3’-UTR mutant was inserted into the pmiR-RB-REPORT™ control vector at the same sites. For reporter assays, BGC-823 cells were co-transfected with wild-type (mutant) reporter plasmid and miR-141-Ribo™ mimic (miR-Ribo™ negative control). Luciferase activity was measured 48 h post-transfection as described previously [17].

### Animal tumor model

Male athymic nude mice (5-6 weeks) were purchased from Shanghai Laboratory Animal Centre (Chinese Academy of Sciences, Shanghai, China). Cultured BGC-823 and MGC-803 cells were transfected with si-MIAT or control vector lentivirus. To generate the orthotopic model, stable infected cells (1 × 10^7^) were injected subcutaneously into the flank region of the nude mice. Tumor diameters were measured every three days, and volumes calculated using the estimation: width^2^ × length× 0.5. Animals were sacrificed on day 24 and tumor weights were measured.

To generate the lung metastasis model, stable infected BGC-823 cells (1 × 10^7^) were injected into tail vein of the nude mice. The mice were sacrificed 5 weeks after the injection. The size and weight of the lungs were assessed, and visible tumors on the lung surface were counted.

### Immunofluorescence

Tumor tissues from mice (5 μm thick) were immunolabeled with anti-DDX5 monoclonal antibody (Cell Signaling Technology; Beverly, MA, USA. 1:100 dilution). Then, dishes were washed and incubated with Alexa Fluor 488-conjugated secondary antibodies (1:50 dilution) for 1 h at room temperature. Nuclei were stained with Hoechst (10 mg/ml) for 2 min. Samples were examined with fluorescence microscope (Zeiss, Oberkochen, German).

### Statistical analysis

Statistical analysis was performed with statistical analysis software SPSS 19.0 software. Statistical analyses were performed using either an analysis of variance (ANOVA) or Student’s *t*-test. Data were expressed as mean ± standard deviation. *P* < 0.05 was considered to be significant.

## Results

### MIAT was up-regulated in GC tissues and cell lines

**Fig. 1:**
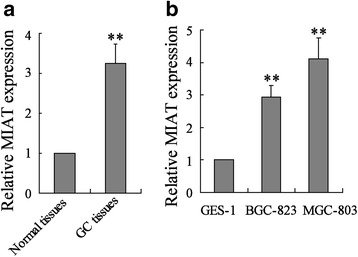
MIAT was up-regulated in GC tissues and cell lines. **a** MIAT expression was measured by real-time PCR in GC tissues and matched adjacent normal tissues (Normal tissues). **b** MIAT expression was measured by real-time PCR in GES-1, BGC-823 and MGC-803 cells. **P < 0.01, compared to normal tissues or GES-1 cells

To determine MIAT expression in gastric cancer tissues, we performed realtime PCR in gastric cancer tissue specimens and matched adjacent normal tissues. The result showed that MIAT level was significantly increased in gastric cancer tissue (Fig. 1a). In addition, we also measured MIAT expression in GES-1, BGC-823 and MGC-803 cells. As shown in Fig. 1b, MIAT expression was significantly up-regulated in BGC-823 and MGC-803 cells compared with GES-1 cells. Similar results were observed in SGC7901 and HGC27 cells (Additional file 2: Figure S1A).

### Upregulation of MIAT was correlated with clinicopathological characteristics in GC patients

We investigated MIAT expression level in 120 patients with gastric cancer and measured their associations with clinicopathological factors. In the present study, the average fold change of MIAT (MIAT in GC tissues compared with matched adjacent normal tissues) was 3.26-fold. The average fold change was used as the threshold, and MIAT expression in GC tissues were separated into high expression (above 3.26-fold) and low expression (below 3.26-fold). As shown in Table 1, the expression level of MIAT in gastric cancer patients were significantly correlated with differentiation degree, TNM stage, lymph node metastasis, and distant metastasis (*p* < 0.05). However, MIAT expression level in gastric cancer tissues was not associated with gender, patient ages, tumor size, and histological type (Table 1).

**Table 1:** Correlation of clinicopathological features of gastric cancer with MIAT expression levels

Characteristics	All cases	MIAT expression levels		
		High (*n* = 65)	Low (*n* = 55)	*P* value
Age				0.512
< 65	62	34	28	
≥ 65	58	31	27	
Sex				0.561
Male	70	38	32	
Female	50	27	23	
Tumor size (cm)				0.108
≤ 5	57	27	30	
> 5	63	38	25	
Differentiation degree				0.004
Well/Moderately	47	18	29	
Poorly	73	47	26	
TNM stage				0.000
I–II	45	15	30	
III–IV	75	50	25	
Histology				0.226
Adenocarcinoma	82	42	40	
Mucinous adenocarcinoma	38	23	15	
Lymph node metastasis				0.000
N0/N1	42	11	31	
N2/N3	78	54	24	
Distant metastasis				0.000
No	92	41	51	
Yes	28	24	4	

### MIAT depletion inhibited GC cell proliferation by cell cycle arrest and apoptosis

**Fig. 2:**
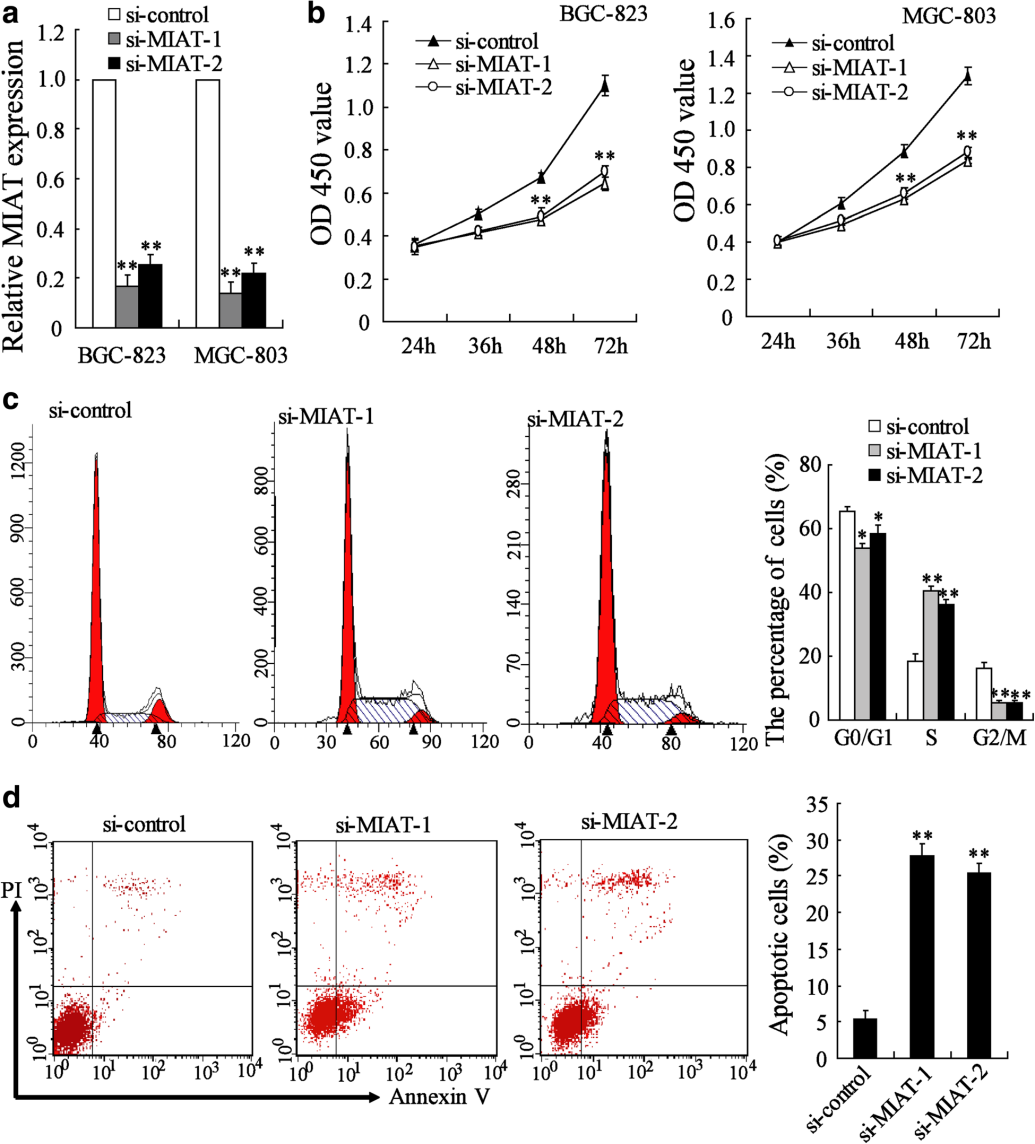
MIAT depletion inhibited GC cell proliferation by cell cycle arrest and apoptosis. **a** BGC-823 and MGC-803 cells were transfected with si-control, si-MIAT-1 or si-MIAT-2 for 24 h, MIAT expression was measured. **b** BGC-823 and MGC-803 cells were transfected with si-control, si-MIAT-1 or si-MIAT-2 for different time, cell viability was measured. **c** BGC-823 cells were transfected with si-control, si-MIAT-1 or si-MIAT-2 for 24 h, cell cycle was determined. **d** BGC-823 cells were transfected with si-control, si-MIAT-1 or si-MIAT-2 for 72 h, cell apoptosis was determined. **P < 0.01, compared to si-control

MIAT was depleted by using small interfering RNA in BGC-823 and MGC-803 cells. The result showed that both si-MIAT-1 and si-MIAT-2 could down-regulate MIAT expression (Fig. 2a). Consequently, cell viability was significantly decreased in si-MIAT-1 and si-MIAT-2 transfected cells than that in si-control transfected cells (Fig. 2b). Next, we determined the effects of MIAT on the cell cycle and apoptosis of gastric cells by flow cytometry. Compared with si-control, si-MIAT-1 and si-MIAT-2 led to an increased proportion of BGC-823 cells in the S phase, but a decreased proportion of cells in G0/G1 phase and G2/M phase (Fig. 2c), indicating that S-phase arrest may be a mechanism of MIAT depletion-induced growth inhibition. Similar results were observed in MGC-803 cells (Additional file 2: Figure S2A). Furthermore, the rate of apoptotic cells in si-control, si-MIAT-1 and si-MIAT-2 transfected BGC-823 cells were 5.47%, 27.7% and 25.33%, respectively (Fig. 2d). Similar results were observed in MGC-803 cells (Additional file 2: Figure S2B). These results suggested that MIAT depletion may inhibit the growth of GC cells by inducing S-phase arrest and apoptosis.

### MIAT depletion inhibited migration and invasion of GC cell

**Fig. 3:**
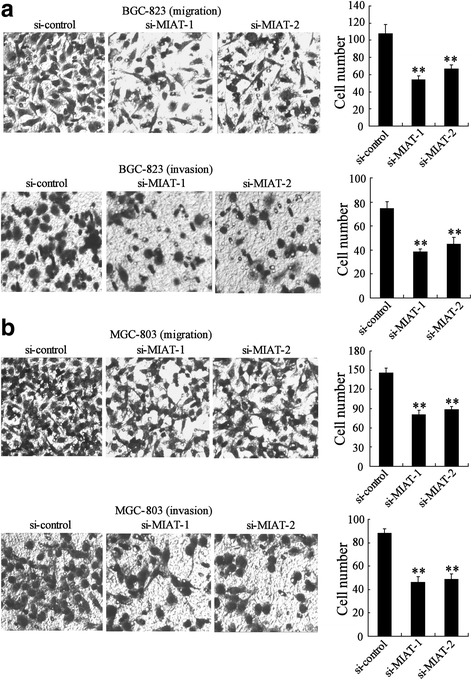
MIAT depletion inhibited migration and invasion of GC cell. **a** BGC-823 and (**b**) MGC-803 cells were transfected with si-control, si-MIAT-1 or si-MIAT-2 for 24 h, cell migration and invasion was measured. **P < 0.01, compared to si-control

We also explored the effects of MIAT on the migration and invasion ability of BGC-823 cells using transwell assays. Results demonstrated that down-regulation of MIAT resulted in a decrease of BGC-823 cell migration and invasion (Fig. 3a). Similarly, migration and invasion was significantly reduced in MGC-803 cell transfected with si-MIAT-1 and si-MIAT-2 (Fig. 3b). These results indicated that that downregulation of MIAT had anti-tumor effect on GC migration and invasion.

### MIAT deletion suppressed GC growth and metastasis in vivo

**Fig. 4:**
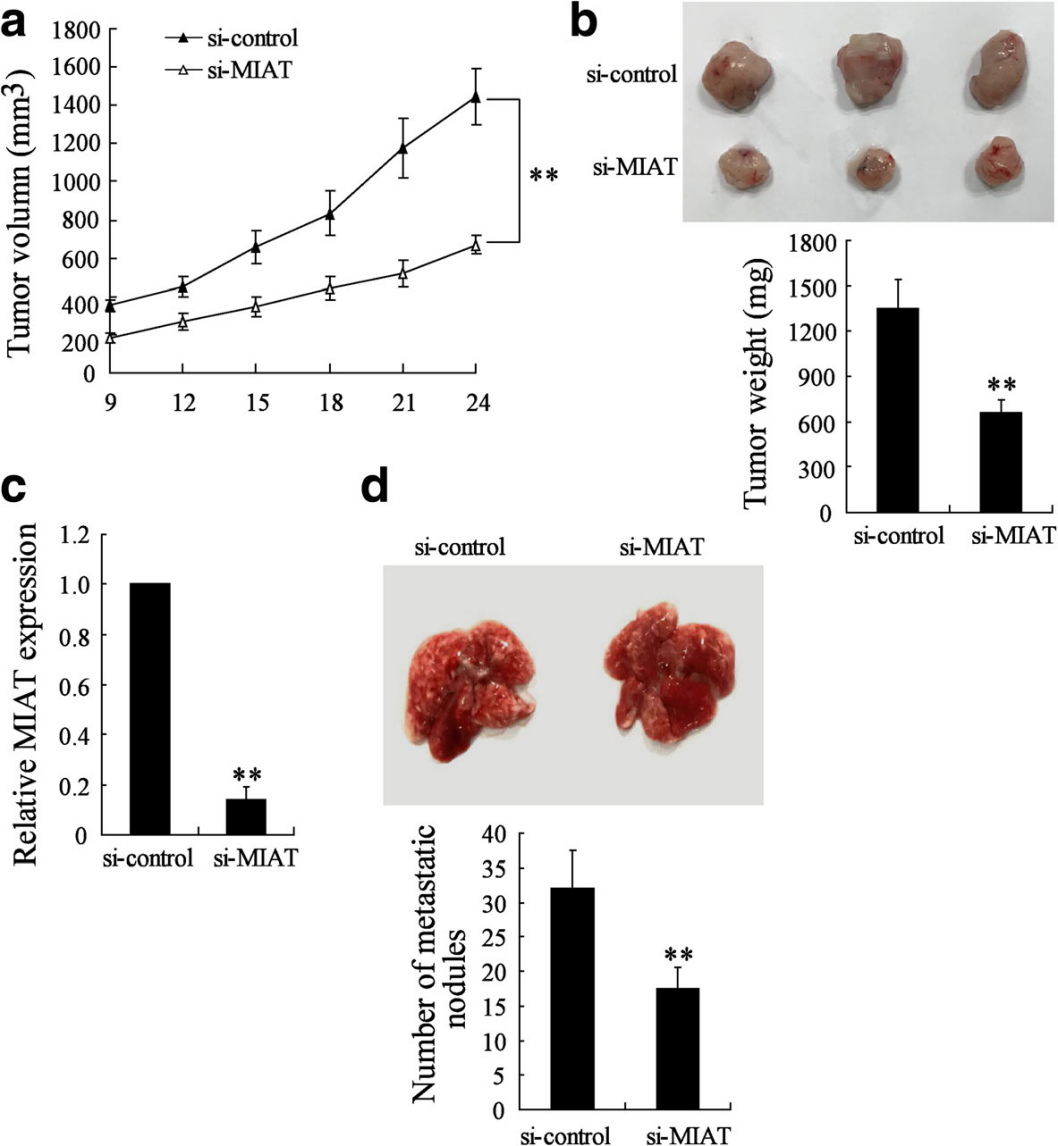
MIAT deletion suppressed GC growth and metastasis in vivo. BGC-823 cells transfected with si-MIAT or control vector lentivirus were injected into the right flank and left flank of nude mice, respectively. **a** Tumor sizes were monitored every three days. **b** After 24 days, images of the tumors were shown and tumor weights were measured. **c** The expression of MIAT in tumor lysates was determined. **d** BGC-823 cells transfected with si-MIAT or control vector lentivirus were inoculated into nude mice via the tail vein. After 5 weeks, the lungs from mice in each experimental group were examined for calculation of the numbers of tumor nodules on lung surfaces. **P < 0.01, compared to si-control

To further investigate the effect of MIAT down-regulation on GC growth in vivo, we established xenograft tumors in nude mice using BGC-823 cells. The tumor volume in si-MIAT lentivirus group was significantly smaller than that in control lentivirus group (Fig. 4a). The average tumor weight in si-MIAT-treated BGC-823 cells xenografts was obviously lower than that in si-control group (655.5 ± 85.39 mg vs. 1353.68 ± 182.62 mg, *P* < 0.01) (Fig. 4b). The down-regulation of MIAT in tumor lysates was also confirmed (Fig. 4c). We also observed that the tumor weight in mice injected with MGC-803 cells transfected with si-MIAT lentivirus was significantly smaller than those in mice injected with MGC-803 cells transfected with control lentivirus (Additional file 2: Figure S3).

Next, we inoculated BGC-823 cells stably depleting MIAT into nude mice via the tail vein to explore the in vivo effects of MIAT on the metastasis of GC cells. Decreased MIAT expression led to a reduction in the number of metastatic nodules when compared with the control group (Fig. 4d). Therefore, our animal experiments demonstrated that MIAT knockdown could inhibit GC growth and metastasis in vivo.

### MIAT acted as miR-141 sponge in GC cells

**Fig. 5:**
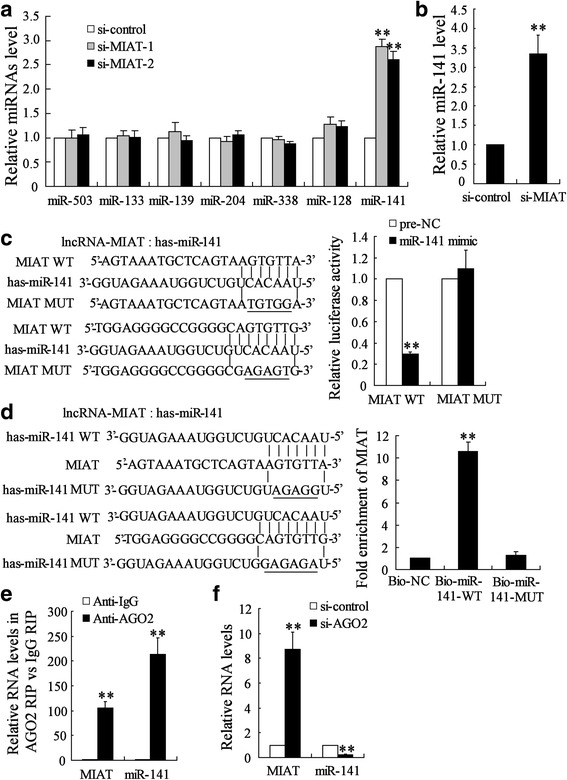
MIAT acted as miR-141 sponge in GC cells. **a** BGC-823 cells were transfected with si-control, si-MIAT-1 or si-MIAT-2 for 24 h, expression of miRNAs was measured. **b** BGC-823 cells transfected with si-MIAT or control vector lentivirus were injected into the right flank and left flank of nude mice, respectively. The expression of miR-141 in tumor lysates was determined. **c** Sequence alignment of miR-132 with the putative binding sites within the wild-type regions of MIAT-1. BGC-823 cells were co-transfected with miR-141 mimic and MIAT-1-WT vector or MIAT-1-MUT vector for 48 h, the luciferase activity was measured. **d** WT and the mutated forms of miR-141 sequence were shown. Detection of MIAT using real-time PCR in the sample pulled down by biotinylated miR-141. **e** The RIP assay was performed to confirm whether MIAT and miR-141 could directly bind to AGO2 in BGC-823 cells. **f** BGC-823 cells were transfected with si-control or si-AGO2 for 24 h. Expression of MIAT and miR-141 was measured. **P < 0.01, compared to si-control, pre-NC, Bio-NC or Anti-IgG

By using a database (starBase2.0), we predicted that 204 miRNAs had matching sites of MIAT. Of those miRNAs, we found that 7 miRNAs (miR-503, miR-133, miR-139, miR-204, miR-338, miR-128 and miR-141), which acted as tumor suppressors in GC, were likely down-stream target of MIAT. We measured expression of these 7 miRNAs in BGC-823 cells by real-time PCR after transfecting cells with si-MIAT-1 and si-MIAT-2. Compared with the expression of si-control, miR-141 expression was obviously increased in BGC-823 cells after transfecting cells with si-MIAT-1 and si-MIAT-2. However, si-MIAT-1 and si-MIAT-2 had no effect on the expression of miR-503, miR-133, miR-139, miR-204, miR-338 and miR-128 (Fig. 5a). Similar results were observed in MGC-803 cells (Additional file 2: Figure S4). Besides, the expression of miR-141 was significantly increased in tumor tissues down-regulating MIAT (Fig. 5b). Luciferase reporter assay showed that upregulation of miR-141 could decrease MIAT-WT activity, but it had no effect on MIAT-MUT (Fig. 5c). In addition, MIAT was pulled down by miR-141, while the introduction of mutations which disrupted the predicted miRNA recognition sites between MIAT and miR-141 resulted in the inability of miR-141 to pull down MIAT (Fig. 5d). These results indicated that MIAT acted as miR-141 sponge in GC cells.

AGO2, a major component of the RNA-induced silencing complex (RISC), plays a key role in mediating miRNA functions [18]. RIP assay showed that MIAT and miR-141 were preferentially enriched in lysate from BGC-823 cells using AGO2 antibody (Fig. 5e). Moreover, Ago2 knockdown led to an increase of MIAT level and a decrease of miR-141 level (Fig. 5f). These data suggested that MIAT acted as miR-141 sponge in an AGO2-dependent manner.

### DDX5 was a novel target of miR-141 in GC cells

**Fig. 6:**
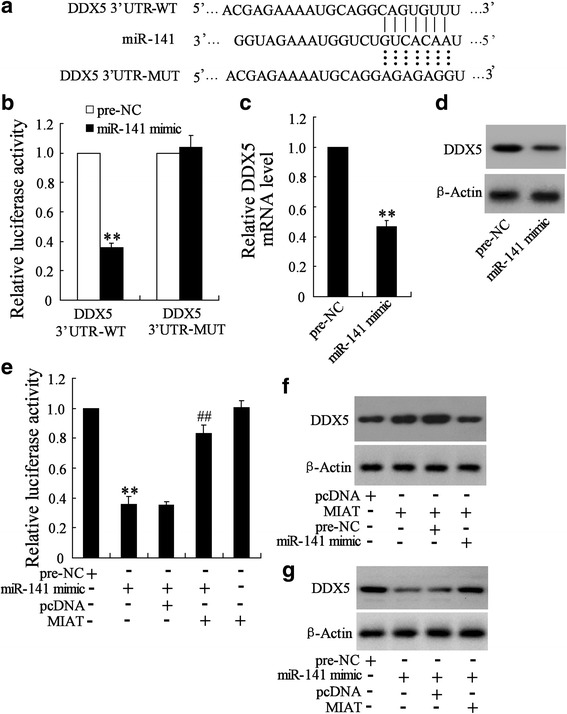
DDX5 was a novel target of miR-141 in GC cells. **a** The 3’-UTR of DDX-5 harbored miR-141 cognate site. **b** BGC-823 cells were co-transfected with miR-141 mimic and wild-type (WT) or mutant DDX-5 3’-UTR for 48 h, the luciferase activity was determined. **c** BGC-823 cells were transfected with pre-NC or miR-141 mimic for 48 h, the mRNA level of DDX-5 was determined. **d** BGC-823 cells were transfected with pre-NC or miR-141 mimic for 48 h, protein level of DDX-5 was determined. **e** BGC-823 cells were co-transfected with pcDNA-MIAT (MIAT), miR-141 mimic and DDX-5 3’-UTR for 48 h, the luciferase activity was determined. **f** pcDNA-MIAT (MIAT) with or without miR-141 mimic were transfected into BGC-823 cells for 48 h, protein level of DDX-5 was determined. **g** miR-141 mimic with or without pcDNA-MIAT (MIAT) were transfected into BGC-823 cells for 48 h, DDX-5 protein level was determined. **P < 0.01, compared to Pre-NC. ##P < 0.01, compared to miR-141 mimic + pcDNA

TargetScan and miRanda showed that the 3’-UTR of DDX5 contained the complementary site for the seed region of miR-141 (Fig. 6a). Further study showed that upregulation of miR-141 significantly decreased DDX5 3’-UTR activity but had no effect on the mutant DDX5 3’-UTR activity (Fig. 6b). In addition, upregulation of miR-141 significantly decreased the DDX5 mRNA and protein expression in BGC-823 cells (Fig. 6c and d). Similar results were observed in MGC-803 cells (Additional file 2: Figure S5A).

To explore whether MIAT competitively suppressed the binding of miR-141 to DDX5, we measure the expression of DDX5 in tumors from mice using immunofluorescence. The result showed that DDX5 expression was significantly decreased in tumor tissues down-regulating MIAT (Additional file 2: Figure S5B). Besides, luciferase reporter assays demonstrated that MIAT could counteract the inhibitory effect of miR-141 on DDX-5 (Fig. 6e). In addition, overexpression of MIAT significantly increased DDX5 level, which was reduced when miR-141 level was upregulated (Fig. 6f). MiR-141 overexpression significantly decreased DDX5 level, whereas reduction was restored when MIAT level was increased (Fig. 6g). Similar results were also observed in MGC-803 cells (Additional file 2: Figure S5C-D). These data suggested that MIAT might act as a competing endogenous RNA to regulate DDX5 expression by sponging miR-141.

### Knockdown of MIAT impaired **GC** cell proliferation and metastasis by inhibiting DDX5 expression

**Fig. 7:**
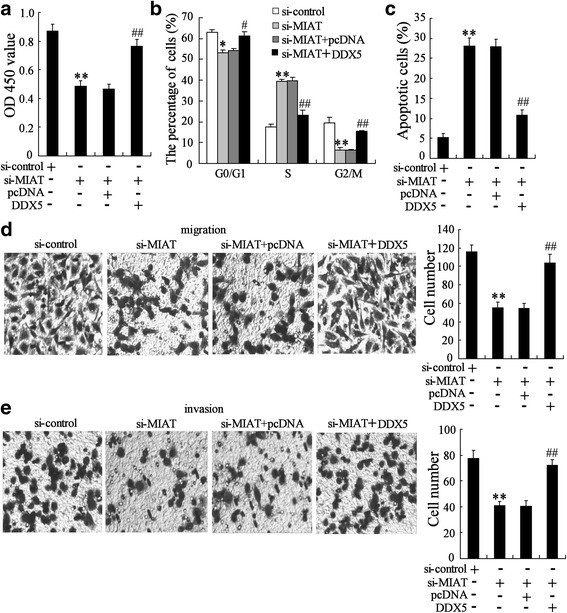
Knockdown of MIAT impaired GC cell proliferation and metastasis by inhibiting DDX5 expression. BGC-823 cells were transfected with si-MIAT-1 and pcDNA-DDX5 (DDX5) for 72 h, (**a**) cell viability, (**b**) cell cycle, (**c**) cell apoptosis, (**d**) cell migration (**e**) and cell invasion were measured. **P < 0.01, compared to si-control. ##*P* < 0.01, compared to si-MIAT + pcDNA

Next, we proceeded to explore whether DDX5 was essential for cell cycle arrest and apoptosis upon MIAT depletion. DDX5 overexpression significantly abrogated the decrease of cell viability on MIAT silencing (Fig. 7a). Furthermore, DDX5 overexpression could restore S-phase arrest in GC cells transfected with MIAT specific siRNA (Fig. 7b). As seen from Fig. 7c, DDX5 overexpression could abolish the increase of apoptotic cells upon MIAT down-regulation. These data strongly indicated that MIAT depletion inducing cell cycle arrest and apoptosis was mainly mediated via downregulation of DDX5 expression.

We also determined whether knockdown of MIAT impaired GC cell migration and invasion by inhibiting DDX5 expression. As expected, DDX5 overexpression could increase migration and invasion in GC cells transfected with si-MIAT (Fig. 7d and e).

## Discussion

Long noncoding RNAs (lncRNAs) have been demonstrated to play important roles in carcinogenesis and development of human GC. LncRNA MIAT has been confirmed to be involved in development and progression of several cancers, such as chronic lymphocytic leukemias, lung adenocarcinoma and prostate cancer [10–12]. However, the role of MIAT in GC has not been investigated. In the present study, we found that MIAT expression was upregulated in GC tissues compared with adjacent normal tissues. In particular, patients with advanced clinical stages, poor differentiation, or metastasis expressed higher levels of MIAT.

To explore the biological function of MIAT in GC progression, we performed in vitro and in vivo assays. Our data showed that MIAT deletion resulted in significant inhibition of cell migration and invasion, while induced cell cycle arrest at S phase and apoptosis in GC cells. Furthermore, our data demonstrated that MIAT knockdown inhibited tumor growth in nude mice xenografts. These data indicated that high expression of MIAT promoted the progression of GC and MIAT may play oncogenic role in human GC progression.

LncRNA can act as competing endogenous RNAs (ceRNAs) to absorb miRNA by sequence complementarity and in turn affecting biological functions of miRNA [19, 20]. Li et al. revealed that MIAT functioned as ceRNAs to activate MAPK signaling pathways by sponging miR-106 in lung adenocarcinoma cells [11]. Initially, we found that MIAT knockdown significantly increased miR-141 level but had no effect on expression of miR-503, miR-133, miR-139, miR-204, miR-338 and miR-128 in GC cells. Then, we used luciferase reporter assays to demonstrate that MIAT contained the miR-141 binding site. Moreover, we verified that MIAT could directly bind to miR-141 and serve as miR-141 sponges in GC cells. Since miR-141 could inhibit proliferation and metastasis of GC [21–23], MIAT exerted oncogenic effect on human GC by negatively regulating miR-141 expression.

DDX5, a transcriptional co-activator of several cancer-associated transcription factors, promoted cancer cell proliferation and metastasis [24]. Previous study demonstrated overexpression of DDX5 promoted GC cell growth, whereas, DDX5 knockdown inhibited cell growth [8]. In the current study, we identified that DDX5 is a novel target for miR-141 in GC cells. Moreover, MIAT and miR-141 appeared to positively and negatively regulate DDX5 expression, respectively. Most importantly, knockdown of MIAT impaired GC cell proliferation and metastasis by inhibiting DDX5 expression. Thus, MIAT/miR-141/DDX5 will provide a novel insight into the mechanism of GC growth and metastasis.

## Conclusions

In conclusion, our study demonstrated that MIAT was upregulated and may function as a ceRNA to increase DDX5 expression by sponging miR-141, which consequently contributed to GC growth and metastasis. Our findings indicated that MIAT could be a potential therapeutic target for GC treatment.

## Additional file

Additional file 1: **Table S1.** Sequences of oligonucleotides used as primers for real-time PCR and siRNA. (DOC 54 kb)

Additional file 2: **Figure S1.** MIAT was up-regulated in GC cell lines. **Figure S2.** MIAT depletion inhibited GC cell proliferation by cell cycle arrest and apoptosis. **Figure S3.** MIAT deletion suppressed GC growth in vivo. **Figure S4.** MIAT deletion regulated miRNAs expression. **Figure S5.** MIAT and miR-141 regulated DDX5 expression. (DOC 2958 kb)

## Abbreviations

DDX5: DEAD-box RNA helicase 5
DMEM: Dulbecco’s modified Eagle’s medium
FBS: Fetal bovine serum
GC: Gastric cancer
MIAT: Myocardial infarction associated transcript
miR-141: microRNA-141
MOI: Multiplicity of infection
Mut: Mutation
PI: Propidium iodide
RIP: RNA immunoprecipitation
si-control: Control siRNA
si-MIAT: Small interfering RNA specific for MIAT
