# Supplementary material for: Long non-coding RNA MIAT promotes gastric cancer growth and metastasis through regulation of miR-141/DDX5 pathway
Source: J Exp Clin Cancer Res. 2018 Mar 14;37:58. doi: 10.1186/s13046-018-0725-3 (PMC5852965; doi:10.1186/s13046-018-0725-3)
Supplement: Supplementary file 1 — Table S1. Sequences of oligonucleotides used as primers for real-time PCR and siRNA. (DOC 54 kb) [file 13046_2018_725_MOESM1_ESM.doc]

**Additional file 1: Table S1**

Sequences of oligonucleotides used as primers for real-time PCR and siRNA.

| Construct |  | Sequences |
| --- | --- | --- |
| Primers for real-time PCR |  |  |
| MIAT | forward | 5'-TTTACTTTAACAGACCAGAA-3' |
|  | reverse | 5'-CTCCTTTGTTGAATCCAT-3' |
| DDX5 | forward | 5'-GCCGGGACCGAGGGTTTGGT-3' |
|  | reverse | 5'-CTTGTGCTGTGCGCCTAGCCA-3' |
| GAPDH | forward | 5'-GGGAGCCAAAAGGGTCAT-3' |
|  | reverse | 5'- GAGTCCTTCCACGATACCAA-3' |
| miR-141 | forward | 5'-GGGCATCTTCCAGTACAGT-3' |
|  | reverse | 5'-CAGTGCGTGTCGTGGAGT-3' |
| miR-503 | forward | 5’-TAGCAGCGGGAACAGTTCTGCAG-3’ |
|  | reverse | 5’-GTGCAGGGTCCGAGGT-3’ |
| miR-133 | forward | 5’-AGTAUUUGGUCCCCUUCAACCAGC-3’ |
|  | reverse | 5’-GTGCAGGGTCCGAGGT-3’ |
| miR-139 | forward | 5'-GCCTCTACAGTGCACGTGTCTC-3' |
|  | reverse | 5'-CGCTGTTCTCATCTGTCTCGC-3' |
| miR-204 | forward | 5'-GTCCCTGTGTCATCCT-3' |
|  | reverse | 5'-CAGTGCAGGGTCCGAGGTAT-3' |
| miR-338 | forward | 5’-TTAGTG TACCAGCCAT-3’ |
|  | reverse | 5’-GAATGCGGGAGCGAA-3’ |
| miR-128 | forward | 5’-CGCGCTCACAGTGAACCG-3’ |
|  | reverse | 5’-GTGCAGGGTCCGAGGT-3’ |
| U6 | forward | 5’-GTGCGTGTCGTGGAGTCG-3’ |
|  | reverse | 5’-AACGCTTCACGAATTTGCGT-3’ |
| Primers for siRNA |  |  |
| si-MIAT-1 |  | 5′-CCAGGCUCCU UUAAACCAATT-3′ |
| si-MIAT-2 |  | 5′-GCAGUUCUUAGCUCAUAUATT-3′ |
| si-DDX5-1 |  | 5’-AACAGGTGCAGCAAGTAGCT-3’ |
| si-DDX5-2 |  | 5’-GAACTGCTCGCAGTACC AA-3’ |
| si-AGO2 |  | 5’-CAGCAGGCACGACTGTGGACACGAA-3’ |
