# Supplementary material for: Long non-coding RNA MIAT promotes gastric cancer growth and metastasis through regulation of miR-141/DDX5 pathway
Source: J Exp Clin Cancer Res. 2018 Mar 14;37:58. doi: 10.1186/s13046-018-0725-3 (PMC5852965; doi:10.1186/s13046-018-0725-3)

**Additional file 2**

**Figure S1 MIAT was up-regulated in GC cell lines.** MIAT expression was measured by real-time PCR in GES-1, SGC7901 and HGC27 cells. **P < 0.01, compared to GES-1 cells.

**
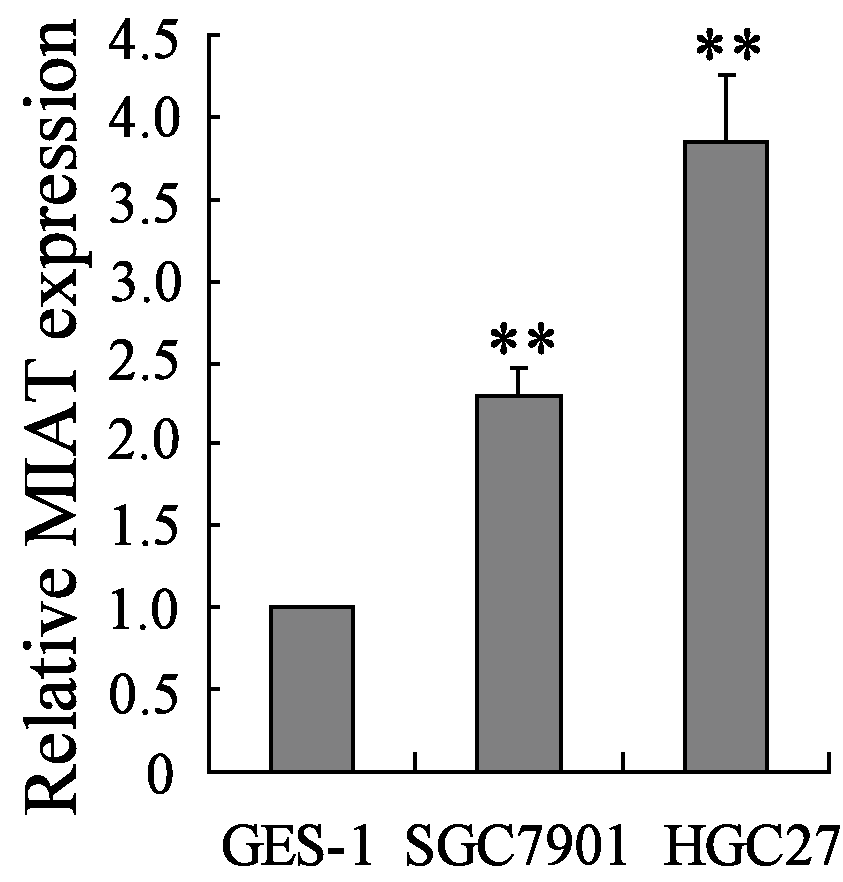
**

**Figure S2 MIAT depletion inhibited GC cell proliferation by cell cycle arrest and apoptosis.** (A) MGC-803 cells were transfected with si-control, si-MIAT-1 or si-MIAT-2 for 24 h, cell cycle was determined. (B) MGC-803 cells were transfected with si-control, si-MIAT-1 or si-MIAT-2 for 72 h, cell apoptosis was determined. **P < 0.01, compared to si-control.

**
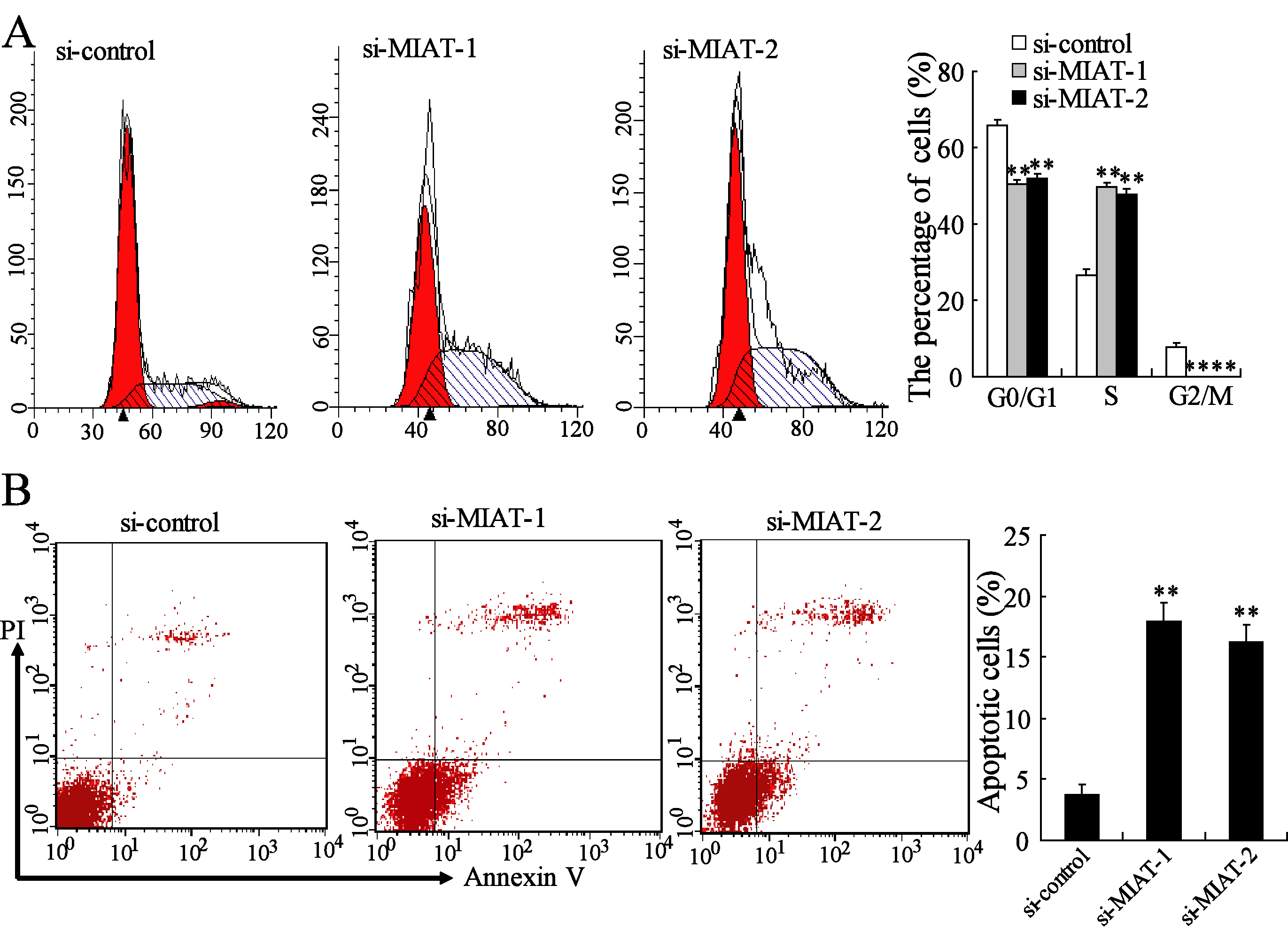
**

**Figure S3 MIAT deletion suppressed GC growth *in vivo*.** MGC-803 cells transfected with si-MIAT or control vector lentivirus were injected into the right flank and left flank of nude mice, respectively. After 24 days, images of the tumors were shown and tumor weights were measured. **P < 0.01, compared to si-control.


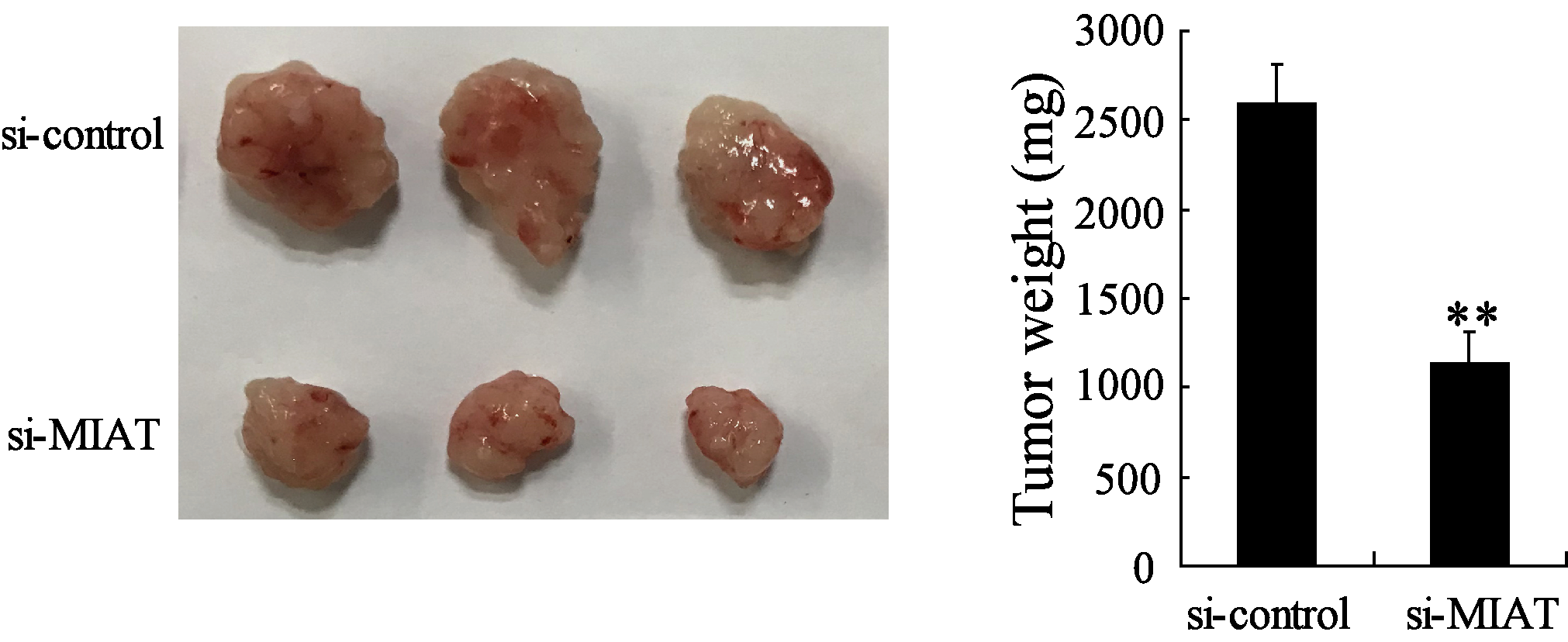


**Figure S4 MIAT deletion regulated miRNAs expression.** MGC-803 cells were transfected with si-control, si-MIAT-1 or si-MIAT-2 for 24 h, expression of miRNAs was measured. **P < 0.01, compared to si-control.


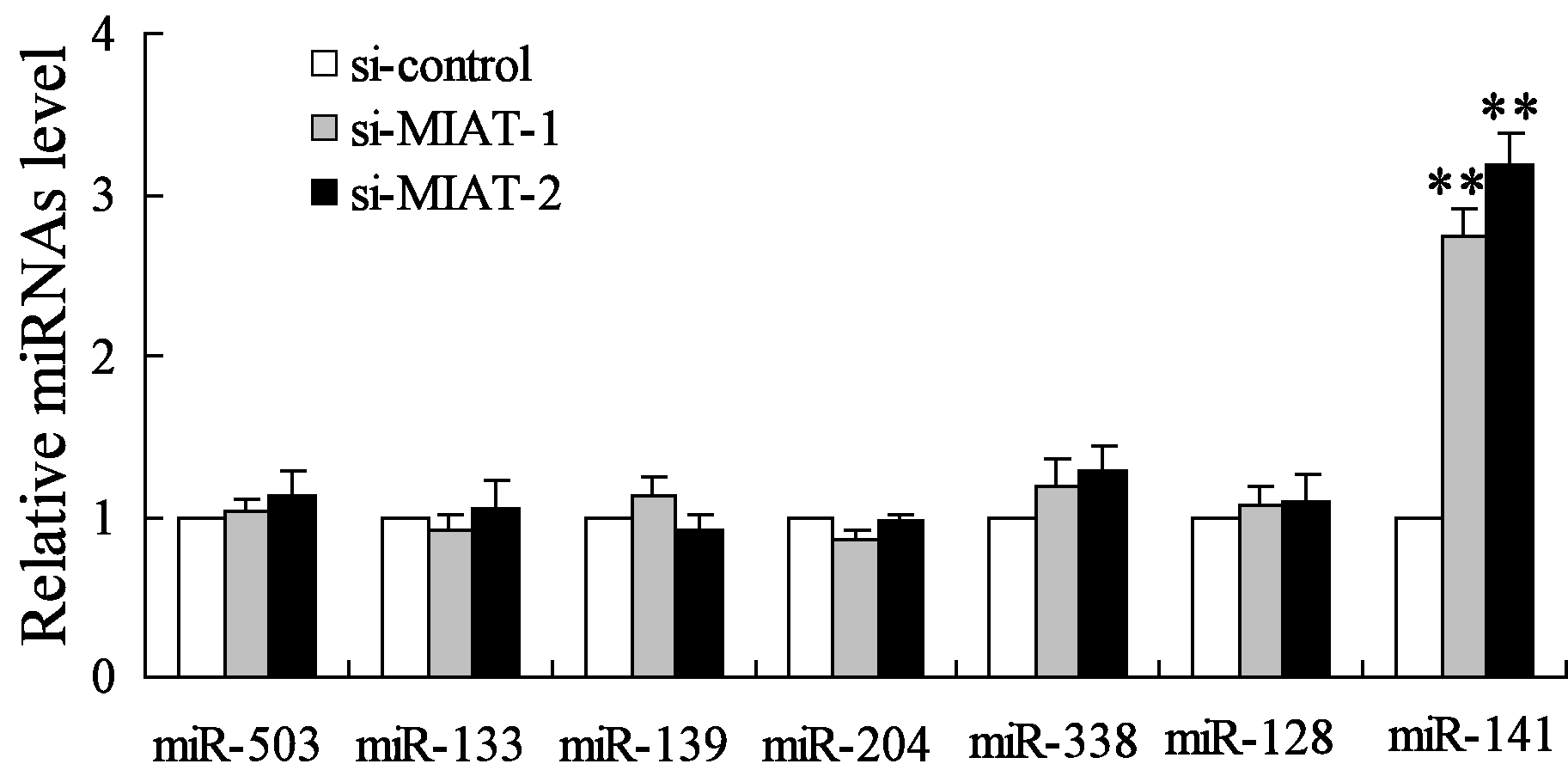


**Figure S5 MIAT and miR-141 regulated DDX5 expression.** (A) MGC-803 cells were transfected with pre-NC or miR-141 mimic for 48h, the mRNA and protein level of DDX5 was determined. (B) BGC-823 cells transfected with si-MIAT or control vector lentivirus were injected into the right flank and left flank of nude mice, respectively. The expression of DDX5 in tumor tissues was determined using immunofluorescence. (C) pcDNA-MIAT (MIAT) with or without miR-141 mimic were transfected into MGC-803 cells for 48 h, protein level of DDX-5 was determined. (D) miR-141 mimic with or without pcDNA-MIAT (MIAT) were transfected into MGC-803 cells for 48 h, DDX-5 protein level was determined. **P < 0.01, compared to Pre-NC.


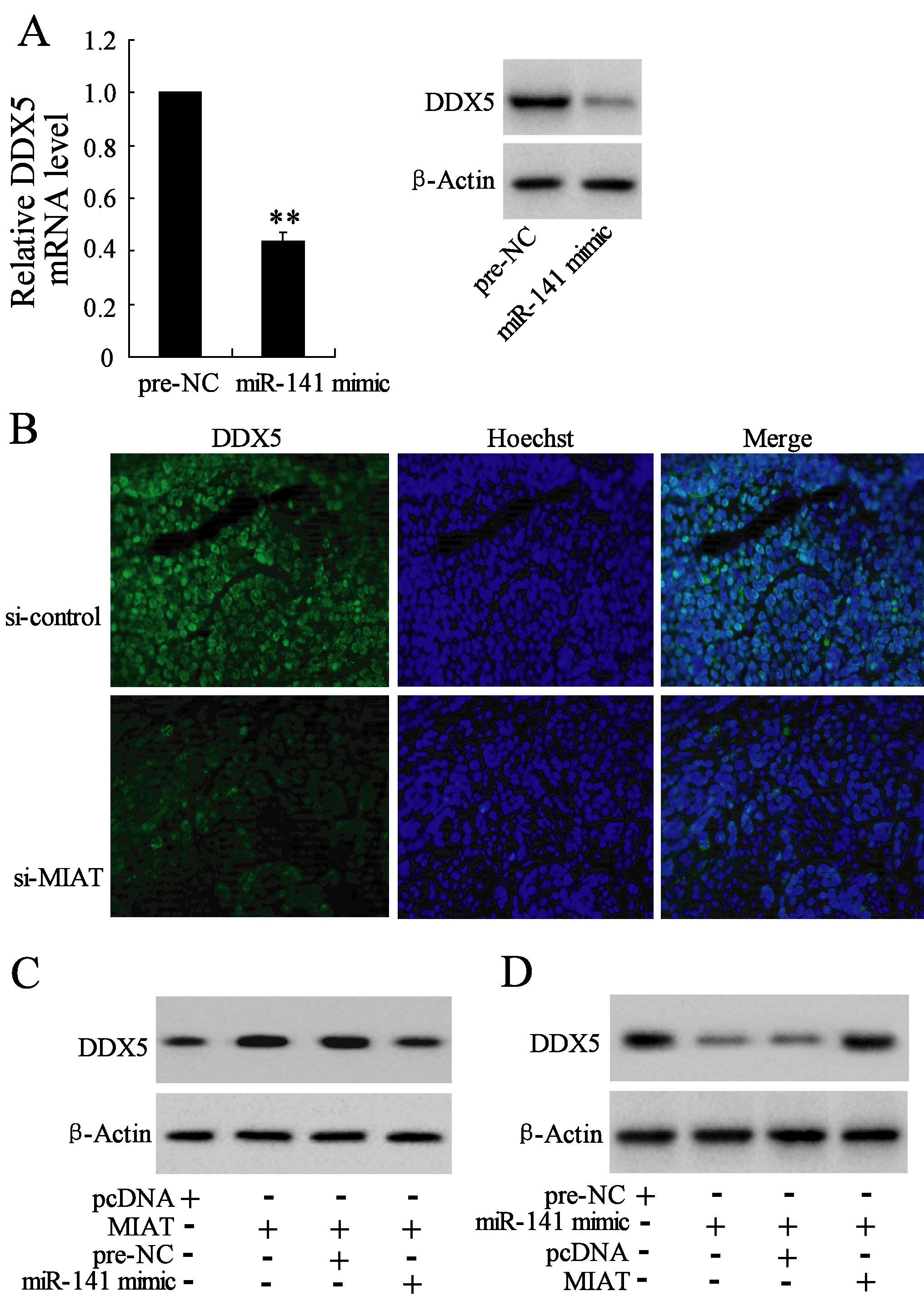

Supplement: Supplementary file 2 — Figure S1. MIAT was up-regulated in GC cell lines. Figure S2. MIAT depletion inhibited GC cell proliferation by cell cycle arrest and apoptosis. Figure S3. MIAT deletion suppressed GC growth in vivo. Figure S4. MIAT deletion regulated miRNAs expression. Figure S5. MIAT and miR-141 regulated DDX5 expression. (DOC 2958 kb) [file 13046_2018_725_MOESM2_ESM.doc]
